# Comparative evaluation of the stability of two different dental implant designs and surgical protocols—a pilot study

**DOI:** 10.1186/s40729-017-0078-2

**Published:** 2017-05-02

**Authors:** David E. Simmons, Pooja Maney, Austin G. Teitelbaum, Susan Billiot, Lomesh J. Popat, A. Archontia Palaiologou

**Affiliations:** 1 Department of Periodontics, Louisiana State University Health Sciences Center School of Dentistry, 1100 Florida Avenue, New Orleans, LA 70119 USA; 2 Tulane University SPHTM, 1440 Canal St, Suite 2001, New Orleans, LA 70130 USA

**Keywords:** Dental implants, Implant stability, OsseoSpeed™, OsseoSpeed TX™, Resonance frequency analysis, Osstell™, Implant survival

## Abstract

**Background:**

The purpose of this study was to compare a parallel wall design implant to a tapered apex design implant when placed in the posterior maxilla using two different surgical protocols.

**Methods:**

Twenty-seven patients (30 implants) were divided into three groups. All implants were 4 mm wide in diameter and 8 mm long.

Group A received 10 tapered implants (OSPTX) (Astra Tech OsseoSpeed TX™) using the soft bone surgical protocol (TXSoft).

Group B received 10 tapered implants (OSPTX) (AstraTech OsseoSpeedTX™) using the standard surgical protocol (TXStd).

Group C received 10 parallel wall implants (OSP) (AstraTech OsseoSpeed™) using the standard surgical protocol (OStd).

All implants were placed in the posterior maxilla in areas with a minimum of 8-mm crestal bone height.

Resonance frequency measurements (implant stability quotient (ISQ)) and torque values were recorded to determine initial implant stability. All implants were uncovered 6 weeks after placement and restored with a functionally loaded resin provisional screw-retained crown. Resonance frequency measurements were recorded at the time of implant placement, at 6 weeks and 6 and 12 months. Twelve months after implant placement, the stability of the implants was recorded and the final restorations were placed using custom CAD/CAM fabricated abutments and cement-retained PFM DSIGN porcelain crowns. After implant restoration, bone levels were measured at 6 and 12 months with standardized radiographs.

**Results:**

Radiographic mean bone loss was less than 0.5 mm in all groups, with no statistically significant differences between the groups. Implant survival rate at 1 year was 93.3%, with 2/30 implants failing to integrate prior to functional loading at 6 weeks. No statistically significant difference was found between ISQ measurements between the three groups at all time intervals measured. Strong positive correlations were found between overall bone loss at 6 months and insertion torque at time of placement. A very weak correlation was found between insertion torque and ISQ values at time of implant placement.

**Conclusions:**

Survival and stability of OSPTX and OSP implants is comparable. Osteotomy preparation by either standard or soft bone surgical protocol presented no significant effect on implant survival and stability for the specific implant designs.

## Background

Dental implants are now a widely accepted treatment option for the replacement of missing teeth. The therapeutic goal of dental implants is to support restorations that replace single or multiple missing teeth so as to provide patient comfort, function, and esthetics as well as assist in the ongoing maintenance of remaining intraoral and perioral structures. However, anatomic limitations such as the maxillary sinus may limit the amount of bone available to place traditional length implants (>10 mm). To avoid invasive sinus elevation procedures, manufacturers have developed shorter implants (<10 mm). Multiple studies have proven that short implants are equally successful to longer implants [1–9]. Tapered implant design further enhances primary implant stability, especially in the posterior maxilla where bone quality is usually poor [10–12].

**Fig. 1:**
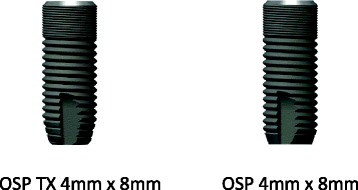
Implant design. The OSPTX and OSP implants are manufactured from high-grade commercially pure titanium with surface roughness produced via a fluoride treatment process. The OSP implant is a screw-shaped self-tapping implant. The diameter used in this study was 4.0 mm. The implant length used in this study was 8 mm. The OSPTX implant has the same features as the OSP except the apex of the implant is tapered

The purpose of this study was to evaluate the initial stability of the OsseoSpeed TX™^1^ tapered implant (OSPTX) and to compare it to the standard OsseoSpeed™^1^ parallel walled implant (OSP) as well as to compare the soft bone and standard surgical protocols. Both implants included in this study are manufactured from high-grade commercially pure titanium with surface roughness produced via a fluoride treatment process. The OSPTX and OSP implants are self-tapping implants. The implants used in this study were all of 4.0 mm in diameter and 8 mm in length. Microthreads™ characterize the coronal aspect of both implants. The OSPTX implant has the same features as OSP except the apex of the implant is tapered (Fig. 1).

Successful integration of dental implants is largely dependent on their primary stability [13]. Implants placed in the maxilla present more challenges due to the poor bone quality usually found in these areas. Another anatomic challenge in the posterior maxilla is the pneumatization of the maxillary sinus which can limit the length of implant that can be placed. To avoid invasive sinus augmentation procedures, implants have been designed in shorter lengths such as 8 mm. To further enhance short implant primary stability, a tapered design has been developed which has been proven to provide greater initial stability [10–12, 14]. Implant stability can be evaluated by different measures such as torque at the time of implant placement, resistance to reverse torque, and resonance frequency analysis (RFA). Multiple studies have established feasibility for validating implant stability in lab and animal models to justify using resonance frequency analysis in clinical trials [15, 16]. Limited literature exists on the OSPTX implant design, and to our knowledge, no clinical studies exist that compare OSP to OSPTX. A recent ex vivo comparison of two different designs of OSPTX implants in porcine mandibles demonstrated that a conical neck design presented higher primary stability (insertion torque and implant stability quotient (ISQ)) than a cylindrical neck design [17]. In our study, both the torque value and ISQ value were recorded at the time of placement. ISQ values were also recorded at implant uncovery at 6 weeks and also at 6 and 12 months when the final restoration was placed.

A recent systematic review by Stocchero et al. concluded that an undersized drilling protocol in soft bone is an effective way to enhance insertion torque but recommended that further clinical studies are needed to confirm these data [18]. Our study was designed to address this question, as it compared the standard drilling protocol to a soft bone protocol.

Our study hypothesis is that the stability of the OSPTX implant will be greater than that of the OSP implant due to the tapered design of the OSPTX implant.

The objectives of this study were the following:

1. To determine whether preparation of the osteotomy with a soft bone protocol (underpreparation of the osteotomy compared to the implant diameter by −0.5 mm at the body portion) results in greater primary implant stability
2. To investigate possible correlations between ISQ and torque values
3. To evaluate radiographic bone loss at 6 months and 1 year

## Methods

Following proper approval by the LSUHSC Institution Review Board (LSUNO IRB#7438), 27 (30 implant sites) systemically healthy patients at least 18 years old were enrolled in the study and randomly divided into three groups as follows (inclusion and exclusion criteria are described in detail in Table 1):

- *Group A* received 10 OSPTX implants using the soft bone surgical protocol (OSPTXSoft).
- *Group B* received 10 OSPTX implants using the standard surgical protocol (OSPTXStd).
- *Group C* received 10 OSP implants using the standard surgical protocol (OSPStd).

**Table 1:** Patient selection criteria

Inclusion	Male or female
	At least 18 years old
	Healthy enough to undergo routine implant surgery and subsequent dental treatment
	Partially edentulous requiring single dental implants in the maxilla
	Adequate volume of native or grafted bone to accommodate dental implants at least 8 mm long
	No active infections
	Physically, emotionally, and financially able to undergo planned implant procedures
	Adequate compliance to meet study requirements and necessary appointments
Exclusion	Medical need for antibiotic premedication for infective endocarditis, artificial joints, or any other medication
	Uncontrolled hypertension
	Uncontrolled diabetes
	Serological human immunodeficiency virus (HIV) positive
	History of significant heart, stomach, liver, kidney, blood, immune system, or other organ impairment or systemic disease that would prevent undergoing the proposed treatment
	Smoke cigarettes or other tobacco products
	Use of investigational drugs during the previous month
	Unresolved dental conditions likely to require exiting the study for treatment, such as deep cavities, abscesses, or moderate to severe periodontal disease
	History of radiation therapy to the head and neck
	Unwilling or inability to sign the informed consent form
	Failure to demonstrate willingness to return for a required number of visits
	Need immediate dental implant placement following tooth extraction

Patient selection, inclusion, and exclusion criteria are presented

To facilitate randomization, the manufacturer packaged each implant with a prescribed surgical protocol included. The surgeon was blinded to the implant type until the opening of the package when the patient was seated for the surgery.

**Fig. 2:**
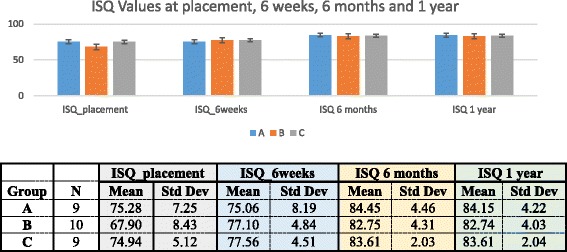
ISQ values at placement, 6 weeks, 6 months, and 1 year. Mean and standard deviation of ISQ values taken at placement, 6 weeks, 6 months, and 1 year is presented. No statistical significant difference was determined between ISQ values at all time points. (*p* < 0.05)

The soft bone drilling protocol used for group A results in an underpreparation compared to the implant diameter by −0.5 mm at the body portion. Corresponding underpreparation at the apex is from the beginning of apex towards the tip of the implant −0.8, −0.4, and 0 mm, respectively. All implants were of 4 mm diameter and 8 mm length and were placed at sites coronal to the maxillary sinus where at least 8-mm bone height was available. Every patient received a cone beam computed tomography (CBCT) evaluation pre-operatively using an i-CAT®^2^ unit. Bone quality was measured clinically by the surgeon during preparation of the osteotomy [19]. Implants were placed following a two-stage protocol. They were uncovered at 6 weeks at which time functionally loaded screw-retained provisional crown was delivered per a FDA approved protocol for this implant system. Implant stability was measured by insertion torque using a calibrated torque wrench^3^ at the time of implant placement and by ISQ measurements using the Osstell™^4^ unit at the time of implant placement and at 6 weeks and 6 and 12 months (Fig. 2). Standardized periapical radiographs were taken at the time of implant placement and at 6 and 12 months. Changes to the bone level heights were measured at 6 and 12 months by two blinded examiners using the ImageJ®^5^ software. The final cement-retained PFM crown (DSIGN porcelain) was delivered at 12 months.

**Fig. 3:**
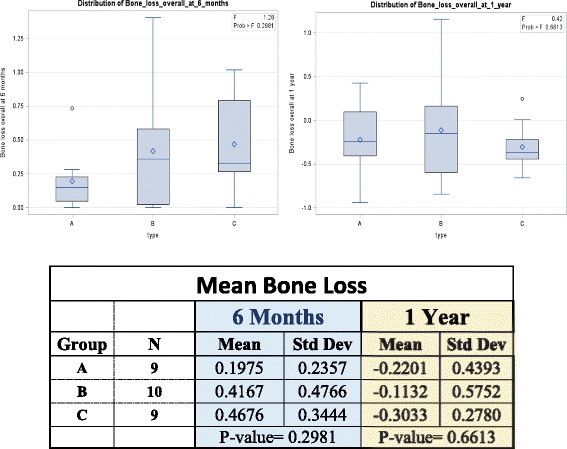
Mean bone loss at 6 months and 1 year. Mean bone loss distribution charts at 6 months and 1 year present no statistically significant difference. *p* value at 6 months was 0.2981 and at 1 year 0.6613

ANOVA was used to compare the mean implant stabilities between the three groups. Post hoc testing was done via Tukey’s honestly significant differences test to calculate the differences between ISQ measurements at the time of implant placement, 6 weeks and 6 and 12 months (Fig. 2) as well as bone levels at 6 and 12 months (Fig. 3). The correlations of multiple parameters such as insertion torque, ISQ, and crestal bone level were calculated using the Pearson product-moment correlation coefficient.

## Results

Overall implant survival rate was 93.3%. Two implants failed, one implant in group A (OSPTXSoft) and one in group B (OSPTXStd). Both implant failures occurred at the time of uncovery (at 6 weeks) and prior to loading of the implants and were attributed to lack of integration. With the exception of these two failed implants, there was 100% success for all remaining implants using the parameters described in Table 2. There are no statistically significant differences in mean crestal bone loss at 6 and 12 months (Fig. 3) or ISQ at insertion, 6 weeks and 6 and 12 months (Fig. 2) in between the three groups. Implant stability, as measured by ISQ, ranged between 83 and 84 at the 12-month time point in all groups (Fig. 2). Mean radiographic crestal bone loss at 6 and 12 months after implant placement was minimal (<0.5 mm) in all groups with no statistically significant difference between the groups (Fig. 3). Implant stability, as measured with ISQ, presented no statistically significant difference between the three groups at the time of insertion and at 6 and 12 months. Strong positive correlations were found between overall bone loss at 6 months and insertion torque at time of placement (*r* = 0.7998). When evaluating the correlation between torque values at the time of implant placement, a strong positive correlation was found with overall bone loss at 6 months (*r* = 0.7995) and with ISQ at 6 weeks (*r* = 0.9078). Insertion torque and ISQ at time of implant placement presented a very weak correlation (*r* = 0.0509).

**Table 2:** Outcome success criteria

Implant success	Clinically immobile when tested manually and/or with RFA (minimum ISQ = 65)
	Absence of peri-implant radiolucency present on an undistorted radiograph
	Absence of unresolved pain, discomfort, infection or neuropathy, or peri-implant soft tissue complications attributable to the implant
	Implant placement that does not preclude delivery of a prosthetic crown with an appearance that is satisfactory to the patient and the dentist
	Crestal bone loss that is <1.5 mm after the first year of loading followed by not more than 0.2 mm of annual crestal bone loss thereafter
Prosthesis success	Absence of unresolved peri-implant soft-tissue complications, such as bleeding, swelling, suppuration or recession, attributable to the prosthetic restoration
	Absence of unresolved prosthetic complications, such as screw loosening or porcelain fracture
	Absence of esthetic complications, such as implant or abutment visibility, or compromised porcelain translucency or mismatched prosthetic tooth color
	Early loading success: a functional provisional crown placed ≥3 weeks and <3–6 months after implant placement, followed by delivery of a definitive crown after 12 months of function

Outcome success criteria are presented

## Discussion

Augmentation of the maxillary sinus prior to dental implant placement is routinely performed in order to help patients restore their maxillary posterior dentition. Unfortunately, not all patients are candidates for this procedure due to either health, personal, or financial concerns. An alternative treatment without the need for a sinus elevation procedure is the use of a shorter implant. Research has shown that shorter implants (<10 mm) have comparable survival and success rates to longer implants (>10 mm) [1–4, 6–9]. Primary implant stability, as measured at the time of placement, is another important factor for both short and long implants. Tapered implant designs are considered to provide greater initial stability [12, 14]. Specifically, Lozano-Carrascal et al. in a prospective clinical study compared OSP implants to tapered MIS® implants placed in human mandibles. They reported the tapered implants achieved higher primary stability measured through ISQ and insertion torque [20]. Our study did not support these findings as we did not find a statistically significant difference in primary stability between the OSP and OSPTX implant designs. However, the OSPTX implants used in our study were tapered only at the apex as opposed to the MIS® implant which is tapered throughout the body of the implant. Furthermore, the mean insertion torque value observed in our study for the OSP group was lower (27.6 Ncm) than that observed by Lozano-Carrascal et al. in the maxilla for the same implant (35.8 Ncm) [20]. This difference may be attributed to the shorter implant length and wider diameter used in our study. The mean ISQ at insertion for the OSP implants in our study presented comparable values to an ex vivo study using the same implant placed in fresh porcine mandibles [17].

Surgical protocols have been developed to overcome the poor bone quality found in the posterior maxilla, so as to increase primary implant stability. Most surgical systems recommend a soft bone surgical protocol which requires a narrower diameter osteotomy than that of the implant being placed. This can involve underpreparing the complete length of the osteotomy or only underpreparing the apical ¾ of the osteotomy when the crestal bone is denser. In the posterior maxilla, the bone quality can vary greatly. By comparing the stability between the three groups, we found that implant stability was neither statistically significantly different between the two different implant designs or between the two different surgical protocols used. These findings are in agreement with Siera-Rebolledo et al., who also found no statistically significant differences between a soft bone drilling protocol and a standard drilling protocol [21].

Insertion torque presented a moderate to strong correlation with ISQ values at 6 weeks, 6 months, and 1 year but not at time of implant insertion. This finding is in agreement with Acil et al. who reported no statistically significant correlation between insertion torque and ISQ at time of implant placement [22].

Although a strong correlation was found between insertion torque and bone loss at all time points, the mean bone loss observed was minimal (<0.5 mm).

The OSP implant system has demonstrated high survival rates ranging from 94 up to 100% in previous long- and short-term studies [23–25]. Our findings are comparable with an overall 93.3% survival rate at 1 year, despite the fact that all implants were placed in the posterior maxilla.

## Conclusions

Survival rates and stability of OSP and OSPTX implants was comparable.

Osteotomy preparation either by the standard or by the soft bone surgical protocol had no significant effect on implant survival, success, and stability.

Insertion torque presented a moderate to strong correlation with ISQ values at 6 weeks, 6 months, and 1 year.

Insertion torque presented a weak correlation to ISQ values at time of implant insertion.

## Footnotes

1 DENTSPLY International, Susquehanna Commerce Center, 221 West Philadelphia Street, York, PA 17401

2 I-CAT 17 19; Imaging Services International LLC, 1910 North Penn Rd., Hatfield, PA 19440

3 Intra-Lock International, 6560 S. West Rogers Circle, Suite 24, Boca Raton, FL 33487

4 Osstell USA, 6700 Alexander Bell Drive, Suite 200, Columbia, MD 21046

5 ImageJ 1.50i Wayne Rasband National Institutes of Health, USA; https://imagej.nih.gov/ij/; Java 1.8.0_77 (64bit)
